# Juvenile Hormone Epoxide Hydrolase: a Promising Target for Hemipteran Pest Management

**DOI:** 10.1038/s41598-017-00907-0

**Published:** 2017-04-11

**Authors:** Abudourusuli Tusun, Ming Li, Xiangzhi Liang, Ting Yang, Bin Yang, Guirong Wang

**Affiliations:** 1 State Key Laboratory for Biology of Plant Diseases and Insect Pests, Institute of Plant Protection, Chinese Academy of Agricultural Sciences, Beijing, China; 2 Department of Entomology, University of California, Riverside, California USA

## Abstract

Juvenile hormone epoxide hydrolase (JHEH) has attracted great interest because of its critical role in the regulation of juvenile hormone (JH) in insects. In this study, one JHEH gene from *Apolygus lucorum* (*AlucJHEH*) was characterized in terms of deduced amino acid sequence, phylogeny, homology modeling and docking simulation. The results reveals a conserved catalytic mechanism of *AlucJHEH* toward JH. Our study also demonstrates that the mRNA of *AlucJHEH* gene was detectable in head, thorax and abdomen from all life stages. To functionally characterize the *AlucJHEH* gene, three fragments of double-stranded RNAs (dsRNAs) were designed to target different regions of the sequence. Injection of 3^rd^ nymphs with dsRNA fragments successfully knocked down the target gene expression, and a significantly decreased survival rate was observed, together with a molting block, These findings confirm the important regulatory roles of *AlucJHEH* in *A. lucorum* and indicate this gene as a promising target for future hemipterans pest control.

## Introduction

The battle between human and pest always faces unexpected problems. In the past decade, transgenic *Bacillus thuringiensis* (Bt) cotton has been successfully used to control the cotton bollworm *Helicoverpa armigera* (Hübner) in China^1^. However, the reduction of chemical insecticides associated with the use of Bt-cotton resulted in frequent outbreaks of the green plant bug, *Apolygus lucorum* (Meyer-Dür), which has become the dominant species in cotton fields in China^2^. So far, calendar-based insecticide spray is the sole management for the control of the green plant bug. Moreover, due to rapid development of insecticide resistance, this bug has become an important pest of Bt cotton and fruit trees in China^3,4^.

In the life cycle of insects, the metamorphosis is regulated by juvenile hormones (JHs) and ecdysteroids. A dramatic decrease in JH titer and a spike in ecdysone titer is usually observed in the final instar of insects. As a result, molting process occurs over a very short period; at the same time, it is very important for the insect development that JH degradation occur quite fast. At least three types of enzymes, JH esterase (JHE), JH epoxide hydrolase (JHEH) and JH diol kinase (JHDK) are involved in JH degradation^5–7^. JHE is a member of the carboxylesterases, secreted in the hemolymph, which hydrolyzes JH to JH acid, which in turn can be reverted to JH^8,9^. Instead, JHEH is a member of the microsomal epoxide hydrolases, which are non-secreted enzymes, active in different organs and tissues. JHEH is responsible for irreversibly opening the epoxide ring of JH to produce JH diol^10,11^. Finally, JHDK catalyzes the phosphorylation of JH diol to produce a more water-soluble metabolite^12,13^.

Because JH is a specific hormone in insects, which plays important roles in the regulation of physiological processes in development and reproductive maturation^14^, interfering with its biosynthesis or degradation process has long been considered a promising strategy for alternative insecticides with low toxicity to non-target organisms^15^. Most of the research on JH degradation has been concentrated on the mechanism of action of JHE, but an increasing number of studies have shown that JHEH is as critical as JHE in insect development, since JH diol is a major metabolite in insects^16–18^. Thus, a characterization of JHEH in the green plant bug is important because of its roles in JH regulation and the irreversible degradation. Based on a recent transcriptome analysis of green plant bug, we have characterized the gene encoding JHEH in *A. lucorum* and, monitored its expression in different life stages. Moreover, we knocked down the expression of JHEH gene by RNA interference (RNAi) and found that the nymphs were not able to develop into next stage and died.

## Results

### Sequence, homology modeling and docking simulations of *AlucJHEH*

**Figure 1:**
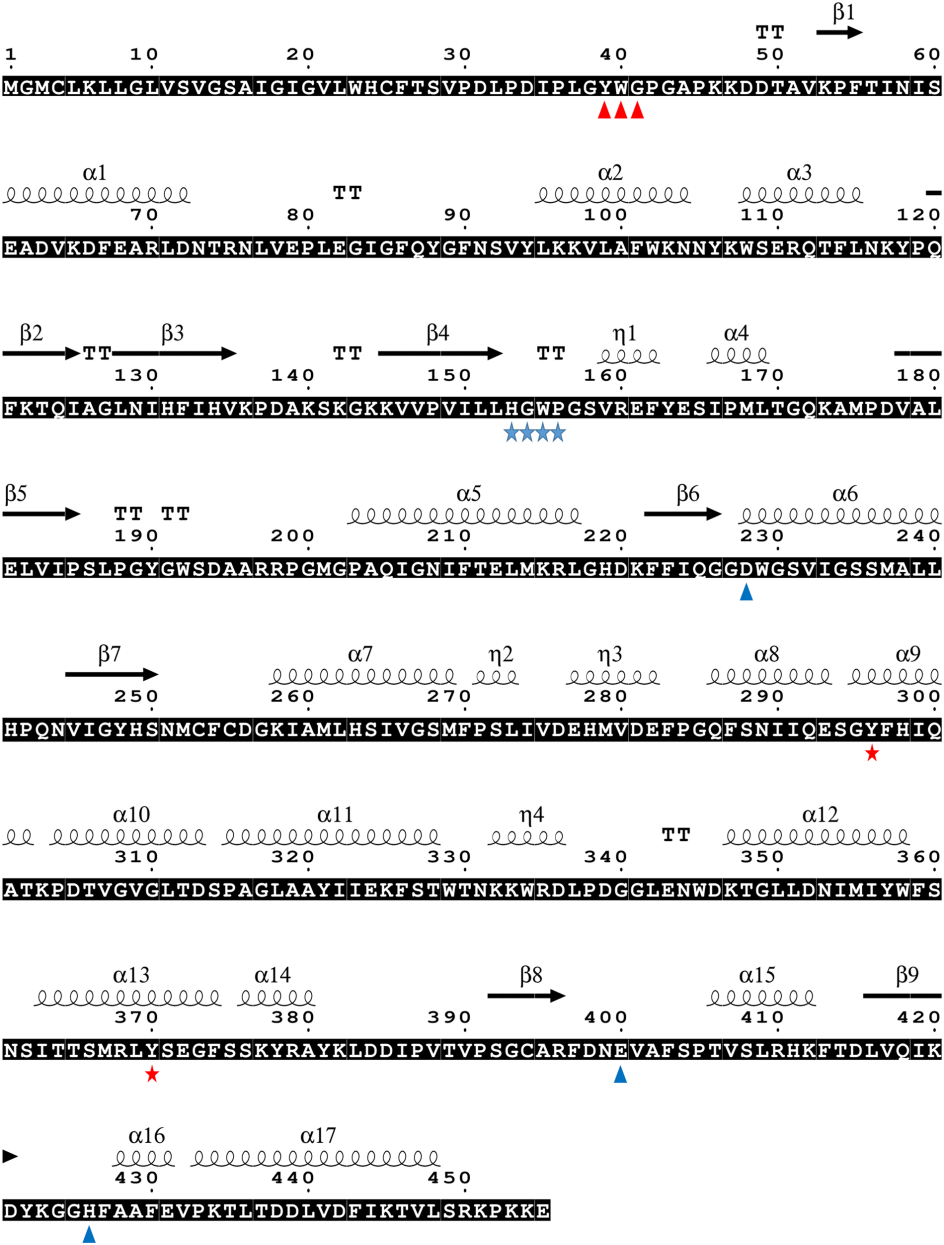
Deduced amino acid sequence of *AlucJHEH*. The sequence analysis was conducted by T-COFFEE (http://tcoffee.crg.cat/) and ESPript 3.0 (http://espript.ibcp.fr/ESPript/ESPript/) (Gouet *et al*. 2003). Alph-helices, eta-helices, beta strands and beta turns are marked by α, η, β and TT, respectively. N-terminal “YWG” anchor motif is marked with red triangle; HGXP motif is marked with blue start; the catalytic triads are marked with blue triangle; two tyrosine residues are labeled as blue start.

**Figure 2:**
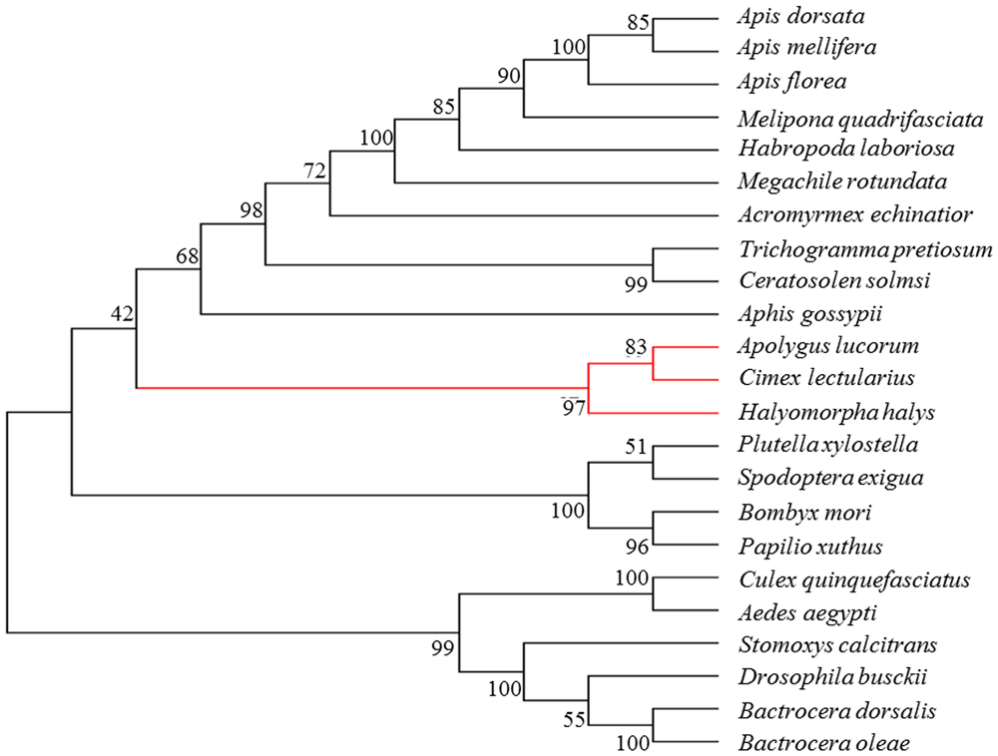
Overall structure of *AlucJHEH*. Alpha/Beta hydrolase domain, Epoxide hydrolase domain, Active site cavity, transmembrane region and active site are colored with cyan, red, purple, green and yellow, respectively.

**Figure 3:**
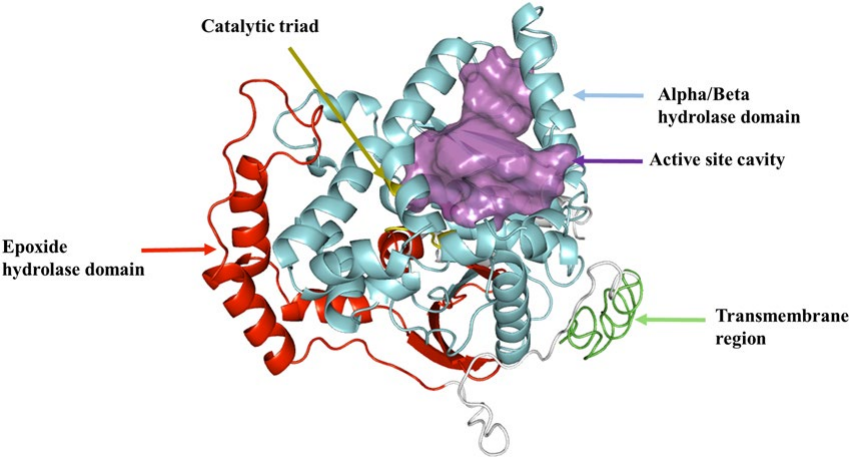
Phylogenetic analysis of JHEH and JHEH-like protein homologs from different insect species. The phylogenetic tree was generated by MEGA 6.0 using the amino acid sequences from *Bombus impatiens* (XP 012240243.1) *Bombus terrestris* (XP 012167590.1) *Melipona quadrifasciata* (KOX80372.1) *Apis dorsata* (XP 006607882.1) *Habropoda laboriosa* (KOC61382.1) *Cerapachys biroi* (XP 011345630.1) *Linepithema humile* (XP 012218175.1) *Solenopsis invicta* (XP 011171353.1) *Vollenhovia emeryi* (XP 011879720.1) *Orussus abietinus* (XP 012286942.1) *Aphis gossypii* (AHW46051.1) *Zootermopsis nevadensis* (KDR10172.1) *Leptinotarsa decemlineata* (AKF11871.1) *Tribolium castaneum* (NP 001161927.1) *Culex quinquefasciatus* (XP 001842664.1) *Aedes aegypti* (AAM88326.1) *Bombyx mori* (BAF81491.1) *Amyelois transitella* (XP 013191892.1) *Helicoverpa armigera* (ACM78602.2) *Papilio machaon* (XP 014361683.1) *Papilio xuthus* (XP 013165848.1) *Halyomorpha halys* (XP 014293302.1) *Cimex lectularius* (XP 014261620.1). Bootstrap values (1000 replicates) are displayed by the nodes.

The open reading frame of *AlucJHEH* gene is 1368 bp long, encoding 455 amino acids, with a calculated molecular weight of 50.78 kDa and isoelectric point (*p*I) of 7.66. *AlucJHEH* contains several conserved signatures, such as catalytic triad (Asp228, Glu400, His426), which is located at the putative active site, an oxyanion hole (HGXP motif with Tyr296 and Tyr370), which stabilizes and donates protons to the oxygen atom of the epoxide ring, and the N-terminal “YWG” anchor motif, involved in subcellular localization (Fig. 1). In addition, a transmembrane region (aa 1–27) at the N-terminus shows that *AlucJHEH* is a membrane-bound protein. Phylogenetic analysis across 23 insect species showed that the *A. lucorum* gene is most similar to those of *Cimex lectularius* and *Halyomorpha halys*, with 60% and 52% identity, respectively (Fig. 2). Homology modeling and JH II docking simulations were performed to investigate the binding properties of *AlucJHEH*. The overall structure of the protein comprises an epoxide hydrolase domain (aa 53–166) an alpha/beta hydrolase domain (aa 180–442) (Fig. 3) and a very large catalytic pocket (1572 Å^3^), suitable for a substrate with long hydrophobic chains, as in JH II.

**Figure 4:**
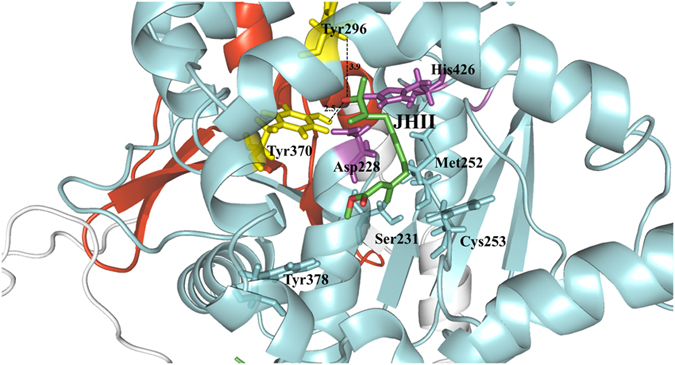
The simulated model of JH II binding to *AlucJHEH*. JH II is shown in green sticks. The residues that binding to the JH II are labeled and shown as sticks.

To further elucidate the JH II binding pattern, docking analysis was performed. Snug fit between the JH II and catalytic pocket of *AlucJHEH* was observed (Figs 3 and 4), suggesting that the active pocket of *AlucJHEH* is ideally shaped for JH II. The epoxide ring oxygen is simultaneously fixed by Tyr296 and Tyr370 via hydrogen bonds, with a short distance (2.3 Å) between the C10 of JH II and Asp228. In addition, the hydrophobic residue, Met 252, and polar residues Ser 231 and Cys 253, which participate in hydrogen bonds formation, were also involved in JH II binding (Fig. 4).

### Expression profile of *AlucJHEH* in different life stages and tissues

**Figure 5:**
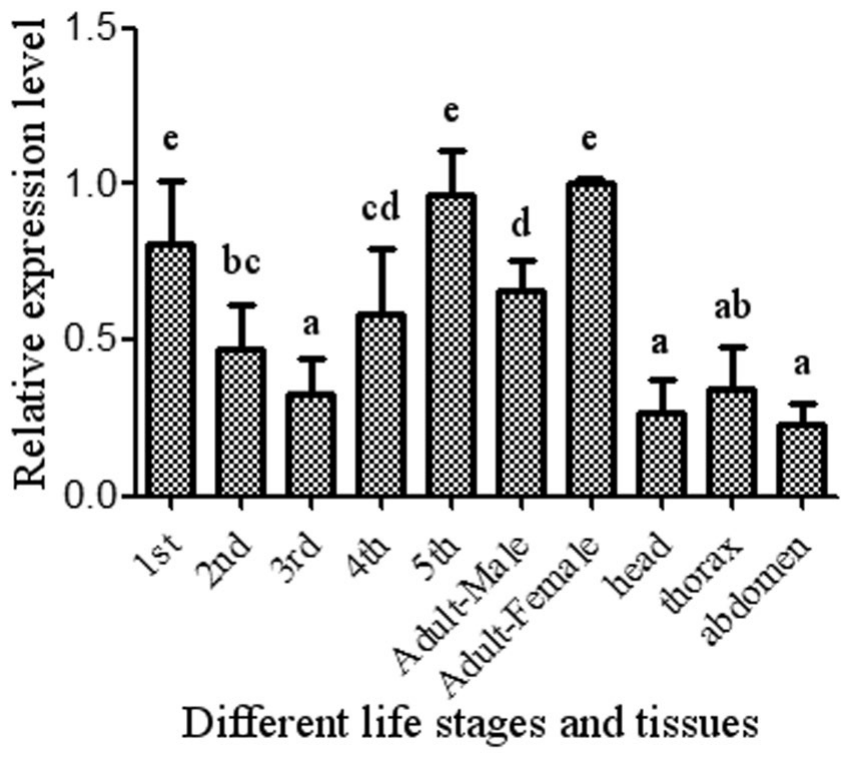
mRNA level of *AlucJHEH* gene in different life stages and tissues. The relative levels of gene expression are shown as a ratio in comparison with that in female adult. The results are shown as the Mean ± S.E. There was no significant difference (*P* ≤ 0.05) in the levels of *AlucJHEH* gene expression among the samples with the same alphabetic letter.

Different expression levels of the *JHEH* gene across all life stages, from nymph to adult, were reported in insects. To monitor the expression pattern of the *AlucJHEH* gene during development and in different tissues of *A. lucorum*, RNA was extracted from tissues (head, thorax and abdomen) of all life stages (1^st^ to 5^th^ instar nymphs and adults) for quantitative real-time PCR. Our results showed that the *AlucJHEH* gene is expressed at different levels in all life stages. In particular it is significantly lower in the 2^nd^, 3^rd^, 4^th^ instar of nymphs and in male adults, compared to the 1^st^ and 5^th^ instar of nymphs and female adults (Fig. 5). When comparing different tissues, we found similar expression levels of *AlucJHEH* gene, with only slightly higher in the thorax.

### Effect of RNAi on the expression of *AlucJHEH* and survival rate of *A. lucorum*

**Figure 6:**
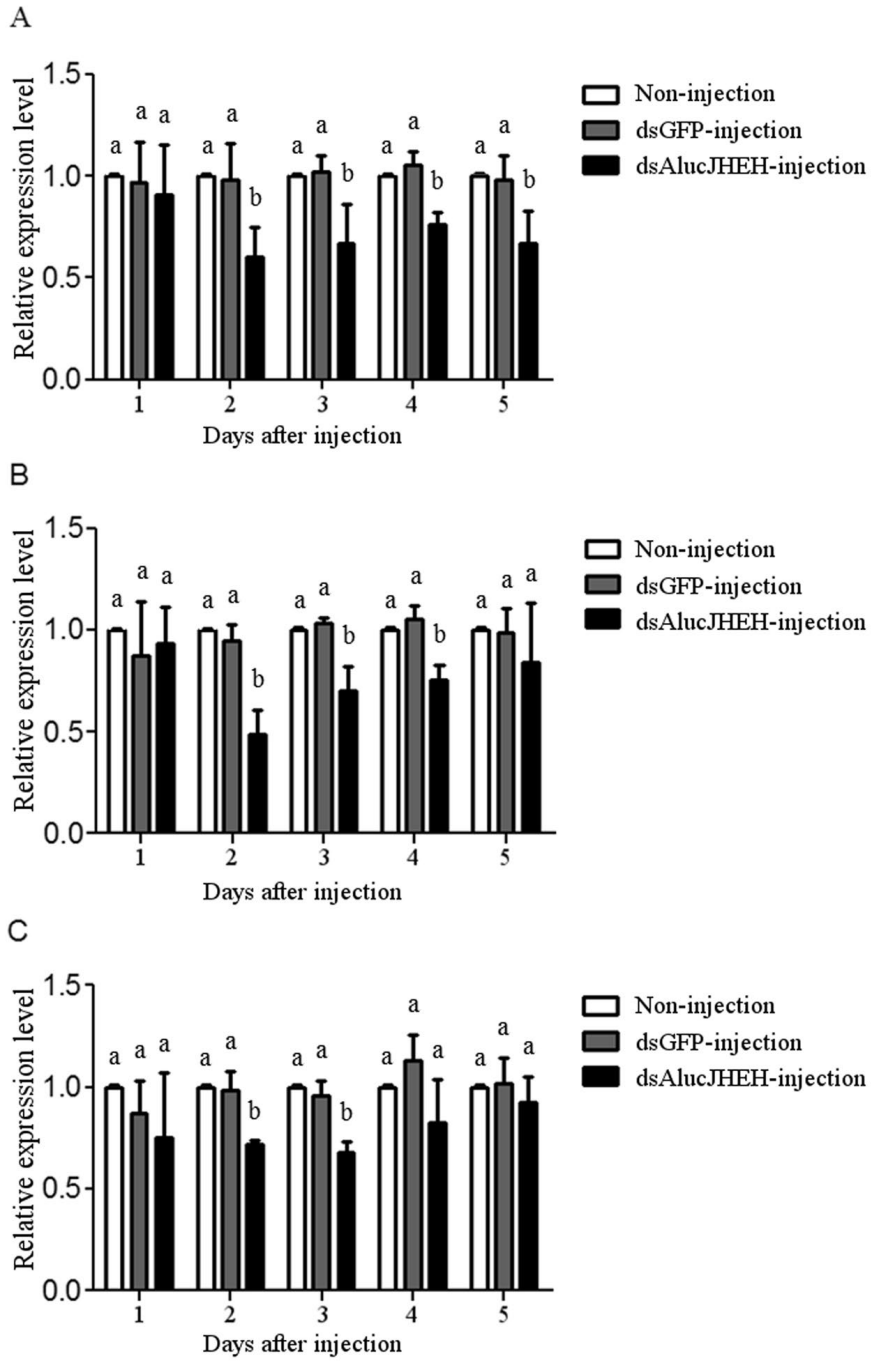
The relative expression levels of *AlucJHEH* gene in *Apolygus lucorum* after injection of different ds*AlucJHEH* fragments. (**A**) Injection of ds*AlucJHEH*-F1. (**B**) Injection of ds*AlucJHEH*-F2. (**C**) Injection of ds*AlucJHEH*-F3. The results are shown as the Mean ± S.E. There is no significant difference (*P* ≤ 0.05) in the levels of *AlucJHEH* gene expression among the samples with the same alphabetic letter.

Three fragments in the sequence of *AlucJHEH* gene (*AlucJHEH-*F1, *AlucJHEH-*F2 and *AlucJHEH-*F3) were selected from the up-, middle- and down-stream regions of for RNAi study. Three dsRNAs (493, 506 and 549 bp) were produced and injected into the body of the early 3^rd^ instar nymphs to determine the role of *AlucJHEH* gene in *A. lucorum* development and molting. On days 2, 3, 4 and 5 after ingestion of dsRNA- *AlucJHEH* -F1, the relative mRNA expression levels of *AlucJHEH* gene were significantly decreased compared with those in the non-injection control and dsRNA-*GFP* injection groups (Fig. 6A). On days 2, 3, and 4 after injection of dsRNA*-AlucJHEH*-F2, the relative mRNA expression levels of this target gene were also found to be significantly decreased (Fig. 6B). However, after injection of dsRNA*- AlucJHEH* -F3 the decrease of mRNA level was observed only on days 2 and 3 (Fig. 6C).

**Figure 7:**
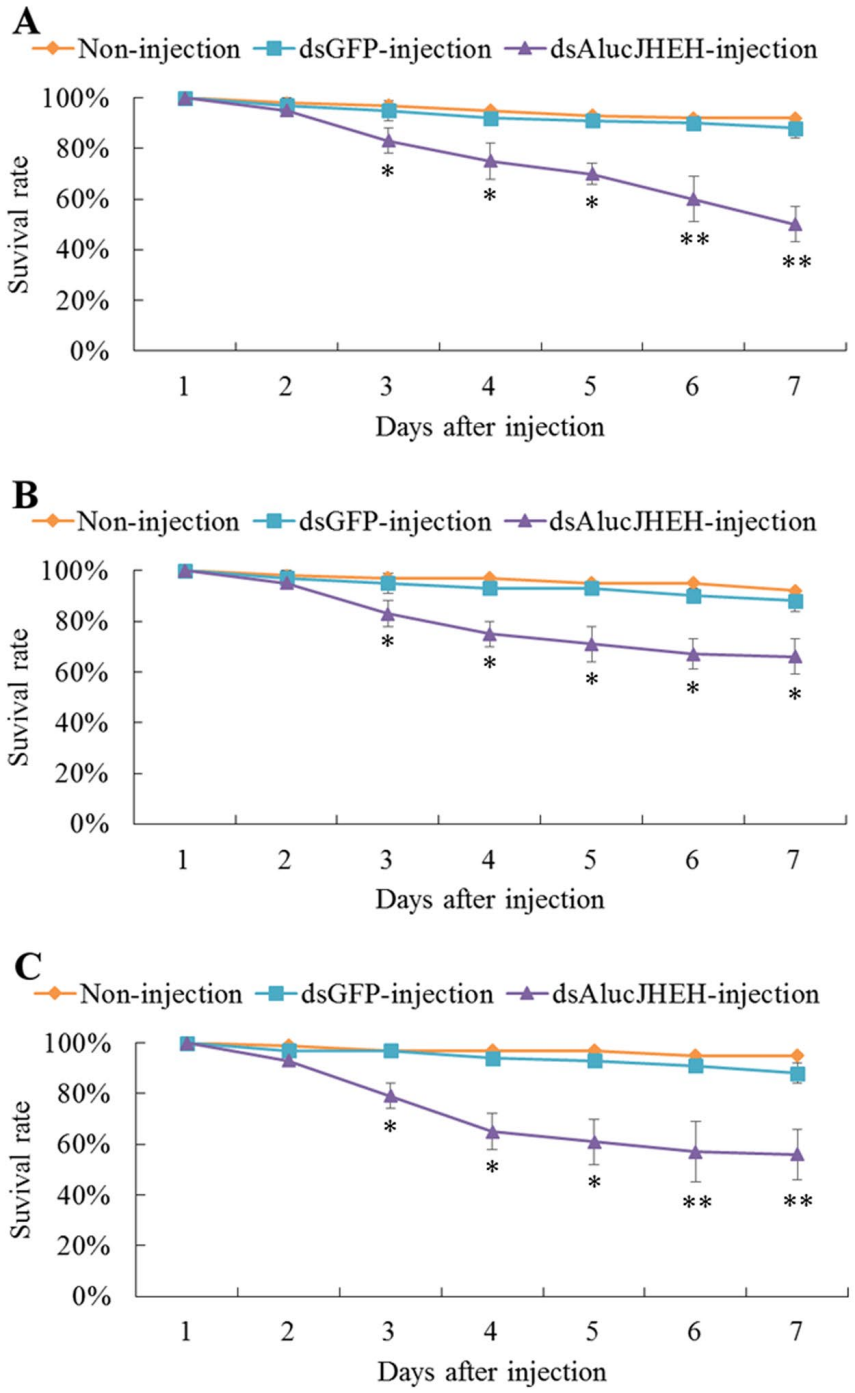
The survival rate of *Apolygus lucorum* after injection of different ds*AlucJHEH* fragments. (**A**) Injection of ds*AlucJHEH*-F1. (**B**) Injection of ds*AlucJHEH*-F2. (**C**) Injection of ds*AlucJHEH*-F3. The results are shown as the Mean ± S.E. *Indicates significant differences in the survival rate between the treated and control groups as determined by *t*-test (*P* ≤ 0.05). **Indicates differences at the p ≤ 0.01 level.

**Figure 8:**
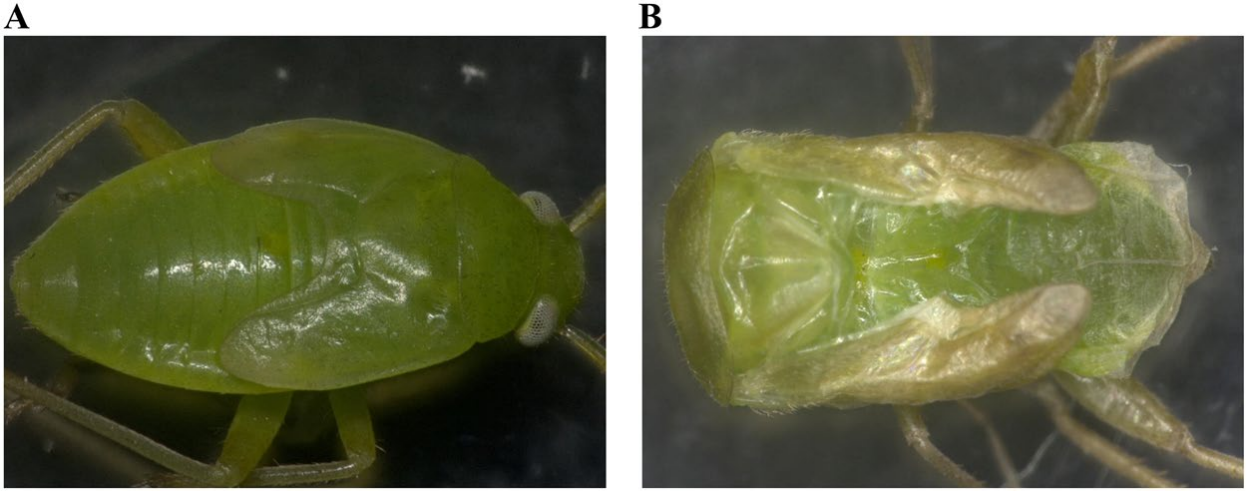
Lethal phenotype produced by injection of ds*AlucJHEH*. (**A**) Control with non-injection. (**B**) The dead green plant bug with injection of ds*AlucJHEH*. The nymph exhibited difficulty in molting and dead finally.

To evaluate the effect of RNAi on the survival rate of the green plant bug, we recorded the number of the living individuals until 7 days post injection. The results showed significant reduction in the survival rate of all three fragments of dsRNA-*AlucJHEH* injection group occurred from 2 days after injection compared with control and dsRNA-*GFP* injection groups (Fig. 7). In addition, some nymphs that were injected with dsRNA-*AlucJHEH-*Fs exhibited difficulty in molting and eventually died. Although apolysis and slippage of the old cuticle was observed, the nymphs were found trapped and dead in the old cuticle (Fig. 8).

## Discussion

JH modulates a variety of development and physiological processes in insects. Previous studies have proved that compared with JHE, which works mainly in the hemolymph, JHEH is a more important regulatory enzyme and could be used to control insect populations, as it irreversibly hydrolyses JH to its diol^19^ (Kumari *et al*.^19^). Based on the transcriptome analysis of *A. lucorum*, only one JHEH gene (*AlucJHEH*) has been detected in this species^20^.

Like all epoxide hydrolases, the *AlucJHEH* protein contains a transmembrane helix, a nucleophile-histidine-acid catalytic triad and “YWG” motif, as well as the “HGXP” motif and two tyrosine residues being part of the oxyanion hole^21^. The modeling and JH II docking simulations showed that *AlucJHEH* contains a hydrophobic substrate binding pocket, and the shape of the pocket could accommodate the molecule of JH II with the epoxide ring exposed to the catalytic residue Asp228, thus indicating that C10 is the nucleophilic attack site by Asp228. This is consistent with other studies showing that the aspartate residue attacks the epoxide ring carbon with the lowest steric hindrance to form a hydroxyl-alkyl-enzyme intermediate^22,23^. Taken together, our results revealed that all of the amino acids involved in the catalytic mechanism are conserved, indicating a common mechanism for the hydrolysis of JH II by *AlucJHEH*.

In order to validate the importance of *AlucJHEH* for the development in *A. lucorum*, the expression profile of this gene in different life stages and tissues was determined by qRT-PCR. As expected, the expression of *AlucJHEH* gene was observed in all life stages and tissues, indicating the importance of this gene. In addition, high expression was detected in the 5^th^ instar nymph and female adults. This result was consistent with other studies that highly expression of *AlucJHEH* decreased the JH titer in the final instar and female of *A. lucorum*, indirectly regulating both metamorphosis and reproduction^24–26^. Interestingly, high expression levels of *AlucJHEH* were also detected in the first instar nymph, since JH is also involved in development, diapause and aging^27,28^, but further studies on the role of *AlucJHEH* in the first instar nymph are needed.

With the extensive use of conventional insecticides (organophosphates, carbamates, organochlorines and pyrethroids) and transgenic Bt cotton, many pests have developed insecticide resistance for most of insecticides, and outbreaks of secondary pests have been recorded. This phenomenon has prompted the development of new compounds with alternative target, model of action, combining high efficiency and selectivity with low toxicity to humans, non-target organisms and environment. Given the important role of JHEH in insect, this gene represents a potential target of pest management, since JHEH is one of the non-secreted key enzymes controlling the JH degradation during insect development, and producing JH diol, which is an irreversibly hydrolyzed product.

Based on the transcriptome analysis of the *A. lucorum*, only one *AlucJHEH* gene has been detected in this species, highlighting the importance of this gene in *A. lucorum* and the potential application of this gene for controlling the population of these insects.

## Materials and Methods

### Insect rearing and sample collection

*A. lucorum* (green plant bug) colony was established and maintained at the Institute of Plant Protection, Chinese Academy of Agricultural Sciences, Beijing, China. The bugs were reared with maize (*Zea mays*) and green beans (*Phaseolus vulgaris*) at 28 ± 1 °C and 60 ± 5% relative humidity (RH) under a photoperiod of 14:10 (L:D) h.

Individuals of *A. lucorum* at different developmental stages, nymph across 1^st^ to 5^th^ instar and two or three day-old adult (male and female, respectively) were frozen immediately in liquid nitrogen, and then stored at −70 °C until use. Tissues of heads, thoraxes and abdomens of 5^th^ instar nymphs were also dissected, frozen and stored at −70 °C separately for future experiments.

### Total RNA extraction and cDNA synthesis

Total RNA of individuals at different developmental stages and different tissues was extracted using TRIzol Reagent (Invitrogen, Carlsbad, CA, USA) according to the manufacturer’s instructions, and then quantitated using a NanoDrop (NanoDrop, Wilmington, DE, USA) and quality assessed by agarose gel electrophoresis. Total RNA from each sample treated with RNase-free DNase I for 30 min at 37 °C to remove residual DNA. First-strand cDNA was synthesized with 1 µg RNA as template and Oligo (dT) 18 primers as anchor primer using Revert Aid TM M-MuLV reverse transcriptase (Fermentas, Glen Burnie, MD, USA) at 42 °C for 60 min. The reaction was terminated by heating for 5 min at 70 °C.

### Double-stranded RNA synthesis

The dsRNAs of the *A. lucJHEH* genes (targets) and a green fluorescent protein (GFP-pMW1650) gene (control) were synthesized by *in vitro* transcription. Firstly, three PCR product fragments (~450 bp) at different regions of the target *A. lucJHEH* gene and one fragment of the control *GFP* gene were obtained using specific primers with T7 promoter sequences (5′-TAATACGACTCACTATAGGG-3′) appended the 5′ ends of both sense and antisense of each PCR product (Table 1). We cloned the PCR product fragments of both target and control genes into the pEASY-T3 cloning vector (TransGen Biotech, Beijing, China) to generate templates for dsRNA production. The respective fragments were amplified by standard PCRs and then purified by using phenol chloroform extraction. Subsequently, dsRNA from *A. lucJHEH* and *GFP* was derived using the T7 Ribomax Express RNAi System (Promega, Madison, WI, USA) with the manufacture’s instruction. The resulting dsRNAs were purified by using phenol chloroform extraction and verified in 1.0% agarose gels. The concentrations were measured using a NanoDrop and adjusted to 10 μg/μL by diluting with nuclease-free water for injection. These dsRNAs were split into 5 μL each and kept at −80 °C until use.

**Table 1:** Primers used in the study.

Primer name	Sequence (5′- 3′)
dsRNA-*AlucJHEH*-F1	TAATACGACTCACTATAGGGGGATGTGTTTGAAGTTGTT
dsRNA-*AlucJHEH*-R1	TAATACGACTCACTATAGGGATGGACTCGTAGAACTCTC
dsRNA- *AlucJHEH*-F2	TAATACGACTCACTATAGGGAAATGGAGCGAACGACAGAC
dsRNA-*AlucJHEH*-R2	TAATACGACTCACTATAGGGCACTATCAACGACGGGAACA
ds RNA-*AlucJHEH*-F3	TAATACGACTCACTATAGGGCGTTGATAGTGGATGAGCACA
ds RNA-*AlucJHEH*-R3	TAATACGACTCACTATAGGGCTTTCTTGGGTTTCCTTGACA
dsRNA-GFP-F	TAATACGACTCACTATAGGGGGAGAAGAACTTTTCACTGG
dsRNA-GFP-R	TAATACGACTCACTATAGGGAGTTGAACGGATCCATCTTC
q-*AlucJHEH*	GCTCAACATTCACTTCATC
q-*AlucJHEH*	ATGGACTCGTAGAACTCT
q-GAPDH-F	TTCCGAGTTCCTGTCCCTAATG
q-GAPDH-R	GCCTCCTTCACCTTCTGCTTG
**q-α-tubulin-F**	**GACTACGGAAAGAAGAGCAAGC**
q-α-tubulin-R	TGCGTCGTCAGAATAGAGTTG

Note: The sequences underlined were T7 promotor sequences. The primers with ds-prefixed were used as ones of dsRNA, and the primers with q-prefixed were used as ones of SYBR-Green qRT-PCR.

### Nymph injection and survival rate analysis

Early 3^rd^ instar nymphs of *A. lucorum* were determined for RNA interference experiment by dsRNA injection. To avoid gene injection affects for target gene expression, dsRNA of the GFP gene were served as the control, and the bugs from the same colony without injection were served as the calibrator. Nymphs were anesthetized with CO_2_ for about 40 seconds and then placed on 1% agarose gel plate with their abdomens upwards under dissection microscope. The dsRNA (~400 ng) was injected vertically to the body axis in the intersegmental membrane region between thorax and abdomen. The manual microinjection procedure was mastered with Nanolatter 2000 (WPI, USA) under slow speed. After injection, the nymphs were immediately removed into new petri dishes, 20 individuals in each, with filter paper at the bottom and fed with fresh corn kernels at normal rearing conditions as described above. A total of 3 replicates were introduced in the experiment. For each replicate, 100 nymphs were applied for each treatment, 60 out of which were recorded daily for survival rate analysis for 7 days, and 40 for sample collection for total RNA extraction to evaluate gene expression levels from 1–5 days post-injection.

### Quantitative real-time PCR (qRT-PCR)

To determine gene expression levels of injected/non-injected bugs and expression profiles of different developmental stages and various tissues, qRT-PCR was performed and analyzed with ABI Prism 7500 Fast Detection System (Applied Biosystems, Carlsbad, CA, USA). Specific primers for qRT-PCR were designed using the Beacon Designer 7.90 software (PREMIER Biosoft International). *GAPDH* and *α-tubulin* genes were used as endogenous control to normalize the target gene expression and to correct for sample-to-sample variation. All samples, including the ‘no-template’ negative control, were performed in triplicate. Each qRT-PCR reaction (20 μL final volume) contained 10 μL 2 x Go Taq qPCR Master Mix (Promega, Madison, WI, USA), 0.5 μL of upstream and downstream primers (10 μM), 1 μL of the sample cDNA and 8 μL of sterilized ultrapure water. Thermocycler program was 95 °C for 2 min, 40 cycles at 95 °C for 30 s, 60 °C for 1 min. The PCR products were then heated to 95 °C for 15 s, cooled to 60 °C for1 min and heated again to 95 °C for 15 s to measure the dissociation curves. Relative expression levels and expression profiles for *A. lucJHEH* gene was analyzed by the 2^−ΔΔCT^ method^29^. Each experiment was repeated at least three times with independent biological samples.

### Phylogenetic analysis

The homologs of *A. lucJHEH* gene were searched for using BlastP from the NCBI non-redundant protein sequences database (http://www.ncbi.nlm.nih.gov/) with the deduced amino acid sequence of *A. lucorum* as the query. Amino acid sequences from 23 insect species were obtained for phylogenetic analysis, including *Bombus impatiens*, *Bombus terrestris*, *Melipona quadrifasciata, Apis dorsata, Habropoda laboriosa, Cerapachys biroi, Linepithema humile, Solenopsis invicta, Vollenhovia emeryi, Orussus abietinus*, from Hymenoptera order; *Aphis gossypii, Halyomorpha halys, Cimex lectularius* from Hemiptera order; Zootermopsis nevadensis from Isoptera order; *Leptinotarsa decemlineata*, *Tribolium castaneum* from Coleoptera order; *Culex quinquefasciatus, Aedes aegypti* from Diptera order; *Bombyx mori, Amyelois transitella, Helicoverpa armigera, Papilio machaon* from Lepidoptera order. And then phylogenetic tree was generated by using the neighbor-joining method implemented in MEGA 6.0.6 software^30^. Branch support was assessed by bootstrap analysis based on 1000 replications. The phylogenetic tree was rendered using FIGTREE v. 1.4 (http://tree.bio.ed.ac.uk/software/figtree/).

### Homology modeling of juvenile hormone epoxide hydrolase

Structural modeling was performed by the I-TASSER server with the combined methods^31,32^. Multiple models were predicted by the I-TASSER for each carboxylesterase. The top scoring model was submitted to the FG-MD server for fragment guided molecular dynamics structure refinement^33^. Model quality was controlled by Ramachandran plots generated with Procheck (http://services.mbi.ucla.edu/SAVES/)^34^ and ProSA-web (https://prosa.services.came.sbg.ac.at/prosa.php)^35,36^. The volume of the substrate binding cavity was characterized by VOIDOO with a 1.4 Å probe^37^. Proteins and ligands were prepared for docking with Autodock Tools v1.5.6 (http://mgltools.scripps.edu/downloads). Molecular docking was performed by Autodock 4.2^38^. Ligand JH II structures were retrieved from the ZINC database (Irwin *et al*.)^39^. For all dockings, a search space with a grid box of 60 × 60 × 60 Å, centered at the aspartate of catalytic triad of *AlucJHEH*. The figures were produced by Pymol (http://www.pymol.org/)^40^.

### Data analysis and graph preparation

One-way ANOVA (P < 0.05) or Student’s t-test (P < 0.05) were applied to determine the significant differences amongst three or more groups, or between two groups, respectively. The graphs of survival rate analysis, expression level and expression profile were plotted in the GRAPHPAD PRISM 5.0 software (GraphPad Software Inc., San Diego, CA, USA).
