# Publisher Correction: Juvenile Hormone Epoxide Hydrolase: a Promising Target for Hemipteran Pest Management

**DOI:** 10.1038/s41598-018-24185-6

**Published:** 2018-04-16

**Authors:** Abudourusuli Tusun, Ming Li, Xiangzhi Liang, Ting Yang, Bin Yang, Guirong Wang

**Affiliations:** 1 State Key Laboratory for Biology of Plant Diseases and Insect Pests, Institute of Plant Protection, Chinese Academy of Agricultural Sciences, Beijing, China; 2 Department of Entomology, University of California, Riverside, California, USA

Correction to: *Scientific Reports* 10.1038/s41598-017-00907-0, published online 11 April 2017

In the original version of this Article, Ting Yang was omitted as a corresponding author. Correspondence and request for materials should also be addressed to tyang@ippcaas.cn. This error has now been corrected in the HTML and PDF versions of the Article.

